# Progression of intervention-focused research for Gulf War illness

**DOI:** 10.1186/s40779-019-0221-x

**Published:** 2019-10-18

**Authors:** Jeremy E. Chester, Mazhgan Rowneki, William Van Doren, Drew A. Helmer

**Affiliations:** 1 War Related Illness and Injury Study Center, Veterans Affairs New Jersey Healthcare System, 385 Tremont Avenue, East Orange, NJ 07018 USA; 2 War Related Illness and Injury Study Center, Veterans Affairs Medical Center, 50 Irving St., Washington, DC, NW 20422 USA; 3 Rutgers University, New Jersey Medical School, 185 South Orange Avenue, Newark, NJ 07103 USA

**Keywords:** Gulf War illness, Chronic multisymptom illness, Gulf War veterans, Gulf War syndrome, Persian Gulf War, Cognitive-behavioral therapy, Exercise therapy, Acupuncture, Coenzyme Q10, Mifepristone, Carnosine, Mindfulness-based stress reduction

## Abstract

The Persian Gulf War of 1990 to 1991 involved the deployment of nearly 700,000 American troops to the Middle East. Deployment-related exposures to toxic substances such as pesticides, nerve agents, pyridostigmine bromide (PB), smoke from burning oil wells, and petrochemicals may have contributed to medical illness in as many as 250,000 of those American troops. The cluster of chronic symptoms, now referred to as Gulf War Illness (GWI), has been studied by many researchers over the past two decades. Although over $500 million has been spent on GWI research, to date, no cures or condition-specific treatments have been discovered, and the exact pathophysiology remains elusive.

Using the 2007 National Institute of Health (NIH) Roadmap for Medical Research model as a reference framework, we reviewed studies of interventions involving GWI patients to assess the progress of treatment-related GWI research. All GWI clinical trial studies reviewed involved investigations of existing interventions that have shown efficacy in other diseases with analogous symptoms. After reviewing the published and ongoing registered clinical trials for cognitive-behavioral therapy, exercise therapy, acupuncture, coenzyme Q10, mifepristone, and carnosine in GWI patients, we identified only four treatments (cognitive-behavioral therapy, exercise therapy, CoQ10, and mifepristone) that have progressed beyond a phase II trial.

We conclude that progress in the scientific study of therapies for GWI has not followed the NIH Roadmap for Medical Research model. Establishment of a standard case definition, prioritized GWI research funding for the characterization of the pathophysiology of the condition, and rapid replication and adaptation of early phase, single site clinical trials could substantially advance research progress and treatment discovery for this condition.

## Background

More than twenty-five years after the Gulf War of 1990–1991, health effects related to the conflict continue to reverberate across the clinical, research, and policy landscape. The Gulf War against Iraq, from August 1990 to April 1991, was fought by coalition forces from thirty-five nations led by the United States (U.S.). This conflict involved the deployment to the Middle East of nearly 1 million coalition troops, including nearly 700,000 Americans. Although the war was temporally shorter than many other historical conflicts, the many exposures that troops encountered during their service in the Persian Gulf likely contributed to medical illness in as many as 250,000 U.S. troops [1]. Beginning in the late 1990s, several epidemiological studies confirmed the existence of a chronic illness, now called Gulf War Illness (GWI), in veterans of the Gulf War, which made clear the need for more research into the pathophysiology, diagnosis, and treatment of GWI [2–7].

GWI is a phenomenon that falls under the umbrella of the broader set of conditions termed chronic multisymptom illness (CMI). CMI has been defined by the Institute of Medicine (IOM) as a cluster of medically unexplained, chronic symptoms that can include fatigue, headaches, joint pain, indigestion, insomnia, dizziness, respiratory disorders, and memory problems [8]. In addition to GWI, other types of CMI include myalgic encephalomyelitis/chronic fatigue syndrome (ME/CFS), fibromyalgia (FM), and irritable bowel syndrome (IBS). When CMI occurs in Gulf War veterans (GWVs), it is more specifically referred to as GWI. Many attempts have been made to further narrow the definition of GWI. In fact, there are at least eight different working definitions for GWI utilized in published studies [9]. Nevertheless, in its 2014 report, the IOM reviewed extant case definitions and found all of them lacking. The panel recommended the use of two case definitions for research: The Centers for Disease Control and Prevention (CDC) definition and the Kansas definition [8].

According to the CDC definition, which is a highly sensitive definition, patients with GWI are GWVs who have had at least one symptom for at least 6 months in 2 of 3 symptom domains (fatigue, pain, cognitive/mood) [2]. Based on the analysis of data on self-reported symptoms from a population-based follow-up survey fielded in 2012, it has been estimated that this definition would classify 50% of GWVs as GWI cases [10].

In 2000, the Kansas case definition was identified empirically as the pattern of self-reported symptoms that best distinguished veterans who deployed to the GW theater of operations from those who did not [10]. It considers a broader variety of symptoms and has both inclusionary and exclusionary components. The Kansas case definition is more specific than the CDC definition because veterans are excluded from consideration as a GWI case if they have been diagnosed by a physician with unrelated chronic conditions that can produce diverse symptoms like those affecting GWVs, or conditions that might interfere with the veteran’s ability to accurately report their symptoms. Additionally, veterans must endorse one moderately severe (scale ranges from mild to severe) and/or multiple symptoms of any severity in at least 3 of 6 symptom domains (fatigue, pain, neurological/cognitive/mood, skin, gastrointestinal, respiratory) to meet the Kansas criteria for GWI. Using the same population-based epidemiologic study of self-reported symptoms conducted in 2012, the Kansas case definition estimates that approximately 34% of GWVs fulfill the GWI criteria [10].

Although it is not known exactly what causes GWI, it has been postulated that the toxic exposures encountered by GWVs are at least partly responsible for their symptoms [11]. The Department of Defense (DoD) estimated that approximately 41,000 service members may have been overexposed to pesticides, approximately 100,000 personnel were possibly exposed to low levels of sarin nerve agent, and another estimated 250,000 ingested pyridostigmine bromide (PB) pills as prophylaxis against perceived chemical weapon exposures [11]. Another prevalent toxic exposure that many troops encountered was smoke from burning oil well fires with the numerous toxic constituents of petrochemical combustion products [12]. Further complicating investigations into a cause, GWI was also reported in an unusually high percentage of veterans of the same era who were not deployed to the war zone [13].

During more than two decades of research, a consensus of the most likely contributing toxic exposures for GWI has emerged, which has shaped the foci of research activities. Many lines of investigation highlight toxic exposures (PB pills, pesticides, and nerve agents) with inhibitory effects against acetylcholinesterase (AChE) [1, 14–17]. Exposure to these acetylcholinesterase inhibitors (AChEIs) may lead to the manifestation of symptoms arising from dysfunction of central and peripheral cholinergic systems. Studies in civilian populations have shown a link between occupational exposure to AChEIs and chronic health symptoms that mirror those of veterans suffering from GWI [18–20]. Neuronal cell death and reduced AChE activity have also been detected in an animal model of GWI consisting of adult rats that were exposed to stress, PB, diethyltoluamide (DEET), and permethrin [21, 22]. Cognitive deficits and mood dysfunction were also observed in the GWI rat models [23–26]. This suggests that exposure to AChEIs may be causally linked to health problems observed in GWVs [11].

Researchers investigated the mechanism of action for AChEI effects in rats and found that AChEI-induced tissue hyperactivity, coupled with AChEI’s concurrent inhibition of oxidative phosphorylation (metabolic pathway in which mitochondria reform ATP), results in a high rate of ATP consumption, compromising the cell’s ability to maintain its energy levels. Thus, it appears that, because of AChEIs exposure, the combination of impaired synthesis of ATP with its greater utilization during tissue hyperactivity results in a significant depletion of ATP [27]. This finding suggests mitochondrial dysfunction as one of the mechanisms underlying GWI. Using these findings as a basis for a new experiment, researchers examined the functionality of mitochondria in veterans suffering from GWI. Their study supported a role for mitochondrial dysfunction and oxidative stress in GWI [28].

Additional emerging evidence suggests a role for chronic inflammation, perhaps mediated by mitochondrial damage and dysfunction, that is particularly damaging to the central nervous system [11, 13, 23, 25, 29–34]. It has been proposed that the neurotoxic effects of AChEIs trigger an inflammatory response that results in tissue damage and dysfunction that produces and perpetuates the chronic symptoms experienced and reported by veterans [6, 14, 35–37]. The mechanism by which AChEIs may induce such an inflammatory response is not well understood. The aforementioned studies focusing on mitochondrial dysfunction and AChEI-induced cell damage implicate reactive oxygen species (ROS)-induced neurodegeneration and muscle tissue damage as the underlying cause of GWI symptoms. A recent study suggests that the AChEI-induced neuroinflammatory response in GWI animal models is independent of acetylcholine (Ach) levels [38].

A related mechanistic theory suggests that AChEI exposure may inhibit microtubule function, disrupting cellular function and contributing to inflammation, especially in neurons. This dysfunction has been demonstrated in animals [22, 39], human and rat cellular models [40, 41] and, indirectly, in ill GWVs in the form of autoantibodies to neuronal components [15]. This nonneuronal effect of AChEI may represent the ‘missing link’ between relatively short-term exposure to the toxic milieu and chronic health effects.

Although there is some evidence supporting a possible link between stress and chronic symptoms in GWVs [42], the role of stress in the etiology of GWI has been controversial. Resistance from some stakeholders in acknowledging the role of stress as a contributory factor has led to some research funding programs explicitly excluding proposals that investigate stress as a causal mechanism of GWI [43, 44]. The current prevailing causal theory retains a possible role for stress, especially in the disability associated with symptoms, but emphasizes the toxic exposures in conjunction with a genetic predisposition among the affected GWVs.

Best practices for the management of GWI are codified by the VA/DoD in a Clinical Practice Guideline for the broader syndrome, CMI, which includes GWI. This guideline recommends several evidence-based treatments for CMI, including graded physical activity, cognitive-behavioral therapy, mindfulness-based therapy, and antidepressants (i.e., selective serotonin reuptake inhibitors (SSRI’s), serotonin and norepinephrine reuptake inhibitors (SNRIs), and mirtazapine). The expert panel relied on a more sensitive, less specific definition of CMI compared to the IOM report from 2014 and acknowledged that while it recommends these practices for GWI, much of the evidence is drawn from different populations, including studies of patients, often civilians, with ME/CFS, FM, and IBS [45].

Although much research has been conducted on GWI, to date, there are few effective or specific treatments for GWI that are strongly supported by evidence. This is a great disappointment and frustrating to patients, healthcare providers, and researchers alike. Although some scientific discoveries are serendipitous, most progress toward efficacious, disease-specific treatments is systematic and incremental [46]. Researchers have estimated that it takes an average of 17 years for new evidence-based findings to reach clinical practice [47]. Only a fraction of eligible patients ultimately receive the benefit of new discoveries because of limited translation into practice. Thus, the National Institutes of Health (NIH) adopted the NIH Roadmap for Medical Research, a framework articulated in 2007 to yield greater translation of treatment into clinical practice. This model describes an idealized progression of research, from basic science to bedside (patient care) to practice-based research to clinical practice, with “translation” occurring between the different phases [48]. The NIH Roadmap also highlights that the flow of scientific knowledge is not strictly unidirectional or linear; for instance, new discoveries made in the process of practice-based research can lead to the development of new basic science theories. Practice-based treatment research refers to dissemination or implementation research supported by postmarketing surveillance, guideline development, meta-analysis, and systematic reviews.

**Fig. 1:**
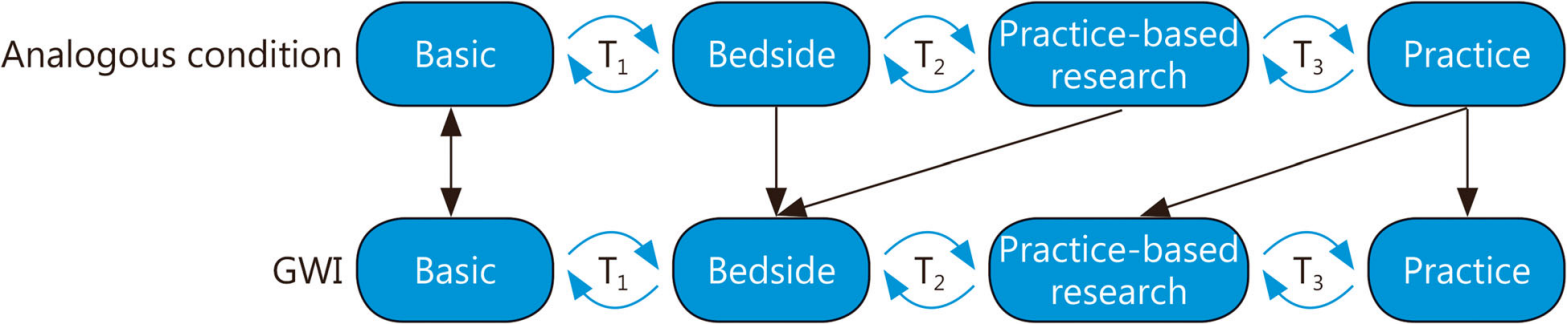
Applying the NIH Roadmap for Medical Research to Gulf War Illness: A collection of far-reaching initiatives designed to transform the nation’s medical research capabilities and improve the translation of research into practice

We consider the NIH Roadmap for Medical Research model here as a framework for a discussion of how GWI intervention research has progressed over the years (Fig. 1). We highlight factors that may have affected the success of different lines of research in achieving the goal of widely utilized, efficacious interventions for veterans with GWI. We identified select treatments for GWI from a systematic literature review of peer-reviewed published and ongoing clinical trials targeting GWI. Here, we present them in standardized form as “case studies” and discuss them relative to the NIH Roadmap for Medical Research model.

Basic: Bench research, preclinical studies, animal research; T_1_: Case series, phase I and 2 clinical trials; Bedside: Human clinical research, controlled observational studies, phase 3 clinical research; T_2_: Guideline development, meta-analyses, systematic reviews; Practice-based research: Phase 3 and 4 clinical trials, observational studies, survey research; T_3_: Dissemination research, implementation research; Practice: Delivery of recommended care to the right patient at the right time, identification of new clinical questions and gaps in care.

## Identification of the literature

### Queried databases

We searched the DoD’s Congressionally Directed Medical Research Programs (CDMRP) and GWI Research Program (GWIRP), the Veteran Administration’s Quality Enhancement Research Initiative (QuERI), and the National Center for Biotechnology Institute (NCBI) PubMed databases for peer-reviewed publications of clinical trials containing at least one of the following keywords: *Gulf War, Gulf War veterans, Gulf War illness, Gulf War syndrome, medically unexplained symptoms,* and *chronic multisymptom illness*: The search results from the PubMed database were further narrowed down by limiting *article types* to “Clinical Trial”. Using our keywords, we also queried ClinicalTrials.gov and the VA’s Office of Research and Development (ORD) and Cooperative Studies Program (CSP), and we did not find any additional clinical trials with published results. The last literature search was performed on June 17th 2019.

### Inclusion/exclusion criteria

**Fig. 2:**
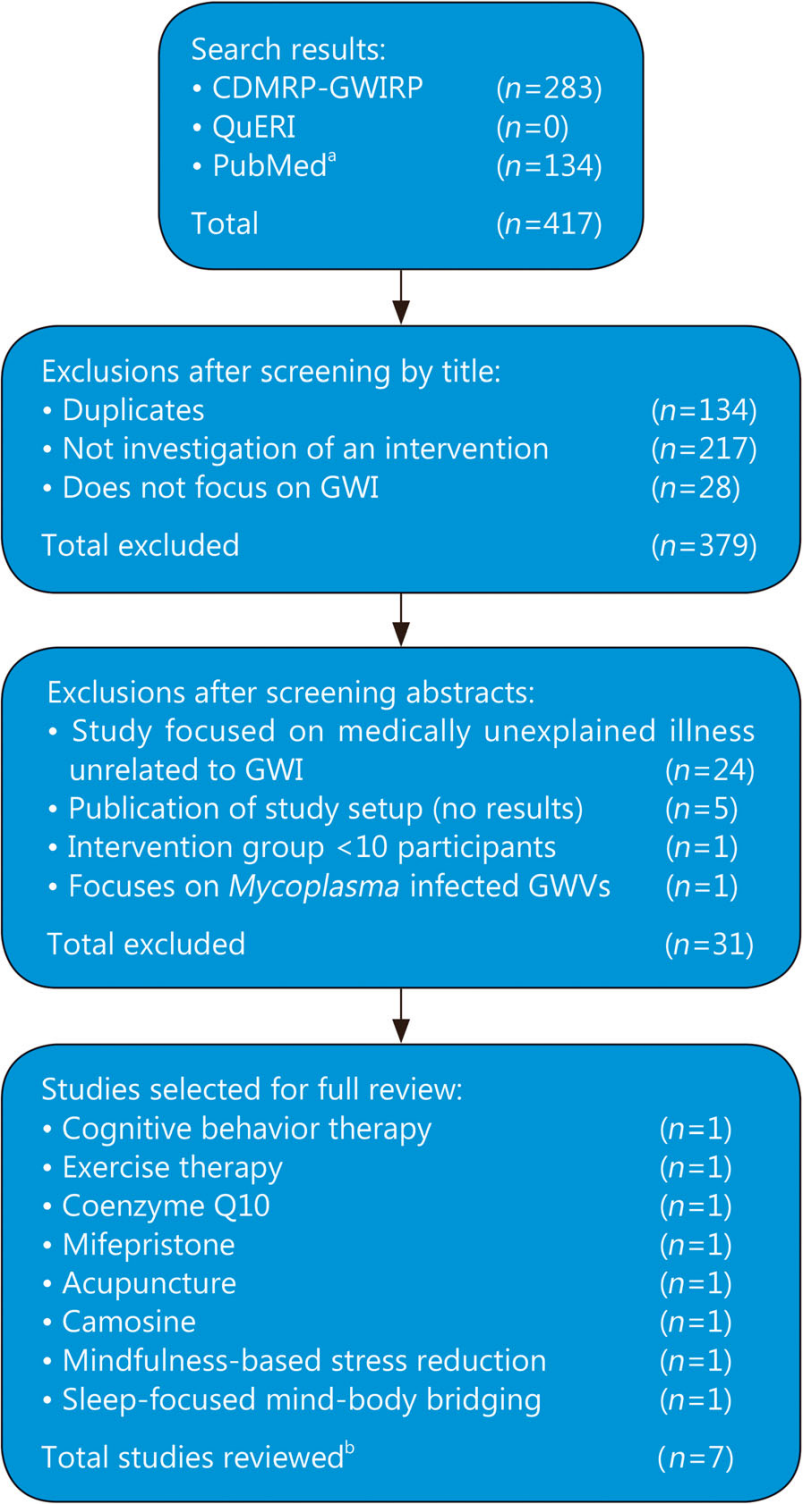
Literature review flow diagram for published clinical trials of Gulf War illness interventions: Methods used identify relevant publications related to Gulf War Illness research. Search keywords: Gulf War illness, Gulf War syndrome, medically unexplained symptoms, chronic multisymptom illness, Gulf War, and medically unexplained illness. ^a^ In addition to use of keywords, the PubMed search results were filtered by limiting article types to clinical trial. ^b^ Cognitive behavior therapy and exercise therapy were investigate in the same study. Thus the total number of fully reviewed studies adds up to 7. GWI. Gulf War illness; QuERI. Quality enhancement research Initiative; GWIRP. GWI research program; CDMRP. Congressionally directed medical research programs; GWVs. Gulf War veterans

We identified 223 studies that met our search criteria. We first screened the search results for relevance by title and then by content in the abstract. The screening criteria used at every step are summarized in Fig. 2. We selected published studies of completed clinical trials involving the administration of an intervention to at least 10 GWVs with GWI. We did not include nonrandomized trials or observational studies in our literature search.

We identified publications on clinical trials of seven distinct interventions (cognitive-behavioral therapy, graded exercise therapy, Coenzyme Q10, acupuncture, mifepristone, carnosine, and mindfulness-based interventions), each of which we discuss below.

We investigated each intervention further by reviewing the associated GWI relevant basic science, clinical research, translational research findings published to date, and any ongoing clinical trials of the intervention in GWI patients. We summarize the rationale and evolution of the investigation of each intervention relevant to its application for GWI.

## Cognitive behavioral therapy

### Description of the intervention

Cognitive Behavioral Therapy (CBT) is a combination psychotherapy in which patients identify and correct maladaptive beliefs (the cognitive component) and utilize thought exercises or concrete actions (the behavioral component) to reduce symptoms and improve functioning [49]. In general, CBT is intended for ongoing, long-term use outside of the therapeutic setting after the specific skills are mastered with a therapist. CBT is an accepted, evidence-based treatment for many disorders, from mood and anxiety disorders (such as depression, panic disorder, and obsessive-compulsive disorder) to insomnia, substance cessation, and pain management.

### History of the intervention

By the late 1990s, CBT had been evaluated in treating ME/CFS and chronic pain syndromes, and it had been found to be efficacious in improving the symptoms and functional status in individuals with these conditions. One randomized clinical trial in 1996 evaluating the efficacy of adding CBT to the medical care of patients with CFS found that 73% of patients receiving CBT in addition to medical care achieved an improvement in functioning (change in Karnofky score by 10 points or more) compared to 27% of patients receiving only medical care (difference of 47% points; 95% CI 24–69) [50]. Another randomized clinical trial in 1997 comparing the efficacy of CBT and relaxation therapy in treating CFS found that 70% of the completers in the cognitive-behavioral therapy group achieved good outcomes (substantial improvement in physical functioning) compared with 19% of those in the relaxation group who completed treatment [51].

### Application of the intervention to GWI

In 1999, researchers investigated the overlap of symptoms and presentation between patients with GWI and patients with ME/CFS and FM [50]. Findings from this study provided the rationale for a randomized controlled trial, funded by the Cooperative Studies Program of the VA and DoD, of 1092 GWVs evaluating the efficacy of CBT and/or aerobic exercise in improving physical function and reducing some of the symptoms of GWI, including pain, fatigue, cognitive symptoms, distress, and mental health functioning. Veterans in this study received usual care (the control group), CBT plus usual care, exercise plus usual care, or CBT and exercise plus usual care for 12 weeks. This study demonstrated that both CBT and aerobic exercise can provide modest relief of some symptoms in GWI. Specifically, this study found that, in 18.5% of participants, 12 months of CBT resulted in a 7-point or greater increase (improvement) on the Physical Component Summary scale of the Veterans Short Form 36-Item Health Survey [52].

### Current and future research in GWI

Ongoing research investigating the use of CBT in the treatment of GWI includes three VA-funded studies: A pilot study evaluating the efficacy of telephone-delivered CBT for veterans with GWI [53], a study evaluating the efficacy of problem-solving therapy (a type of cognitive therapy) in reducing disability in veterans with GWI [54], and a study comparing the efficacy of cognitive therapy alone and sleep restriction alone (a type of behavioral therapy) in treating GWVs with insomnia [55]. Neuropsychological testing or biomarkers (such as C-Reactive Protein, which is elevated in inflammation and which may be elevated during chronic insomnia) may be targets of future investigation, and these are included as secondary outcomes in the ongoing studies.

### Concordance with the NIH model

The use of CBT for treating GWI arose from its demonstrated efficacy in conditions that are considered similar to GWI. The target of treatment is reducing symptom burden and improving function, not necessarily addressing a unique, underlying pathophysiology. There was no explicit basic science or animal model research of relevance conducted in the progression of CBT as a treatment for GWI, although considerable research related to cognition, behavior and the use of CBT has been done in animals and humans [56].

## Exercise therapy

### Description of the intervention

Exercise, a structured and focused form of physical activity, has long been known to confer significant health benefits for many organ systems and in patients with many different illnesses [57]. Research has identified specific, efficacious exercise prescriptions based on age, health status, gender, body habitus, disease/condition, and other factors.

### History of the intervention

Exercise has been shown to be an effective treatment for improving the symptoms of CMI. Peters et al. performed a randomized controlled trial of 228 patients with medically unexplained symptoms, which showed that using aerobic exercise, in comparison to non-aerobic stretching, for 1 hour twice a week for 10 weeks, improved functional outcomes for patients. Improvement of functional outcomes in this study was assessed based on the Hospital Anxiety and Depression Scale (HADS), SF-36 scales, and somatization scales [58]. In a systematic review evaluating five randomized controlled trials involving 150 patients with FM, it was found that performing strengthening exercises twice per week (for 12–21 weeks) resulted in significant improvements in pain and in overall disability (compared to patients in the control groups) [59]. Finally, in a large systematic review of 33 randomized controlled trials involving 2266 patients with FM symptoms, patients who engaged in aerobic exercise were shown to have improvements in pain, quality of life, fatigue, and sleep problems, and the aerobic exercise group had fewer treatment drop outs compared to control patients, who received noninterventions, drug placebos, sham interventions, and minimally active controls such as education and relaxation [60].

### Application of the intervention to GWI

The efficacy of exercise in other CMI populations led to its application as an investigative treatment for veterans with GWI in the late 1990s. From 1999 to 2001, a phase 3, 2 × 2 factorial study funded by the VA and DoD was performed in 1092 Gulf War era veterans over 24 months evaluating the use of CBT and aerobic exercise to treat symptoms of pain, fatigue, and cognitive dysfunction. We previously discussed the results of one arm of this study in the case analysis of CBT as an intervention for GWI. This study also found that at 1 year after beginning treatment, exercise therapy conferred modest improvements in some symptoms, such as fatigue (adjusted mean change from baseline in the Multidimensional Fatigue Inventory score of 2.33 versus − 1.03 for usual care), cognitive symptoms (adjusted mean change from baseline in Cognitive Failures Questionnaire of 2.98 vs − 0.67 for usual care), and mental health functioning (adjusted mean change from baseline in SF-36 Mental Health Index of 3.27 vs − 1.60 for usual care). However, it did not confer significant improvement in physical functioning (adjusted odds ratio of 1.07, 95% CI, 0.76–1.50, for > 7-point improvement in the Physical Component Summary Score on SF-36) or pain (adjusted mean change from baseline in McGill Short-Form pain level of − 0.48 vs − 0.11 for usual care) [52]. Another VA-funded study found that Gulf War veterans with chronic musculoskeletal pain perceive acute exercise as more painful and effortful than healthy Gulf War veterans and experience increased pain sensitivity following exercise [61].

### Current and future research in GWI

We were unable to identify any currently active research trials investigating the effects of exercise on veterans with GWI.

### Concordance with the NIH model

The use of exercise therapy for treating GWI somewhat adheres to the NIH roadmap model. The use of exercise therapy is based on evidence of the benefits of exercise for many health problems and on several large clinical and observational trials in using exercise to treat CMI generally and FM specifically. However, there is limited clinical research (no pilot studies; only one large, phase 3 study) examining exercise therapy to treat GWI specifically. There has been no basic science linking the pathophysiology of GWI to the potential benefits of exercise.

## Mindfulness-based interventions

### Description of the intervention

Two Mindfulness-based Interventions (MBIs) have been tested in treating GWVs: Mindfulness-based stress reduction (MBSR) and Mind-body bridging (MBB).

MBIs are programs that incorporate mindfulness, the psychological process of bringing one’s attention to experiences occurring in the present moment, to assist people with a range of conditions and life issues. MBSR uses a combination of meditation, body awareness, and yoga to help people become more mindful [62], while MBB aims to enhance psychological flexibility and resilience through mental training that promotes recognition and diffusion of negative thoughts and self-centered expectations that increase distress [63]. In recent years, MBIs have been the subject of numerous controlled clinical research trials with evidence suggesting that they may have beneficial effects, including stress reduction, relaxation, reduction of dysfunctional thinking, and improvements to quality of life, but they neither help prevent or cure disease [64].

### History of the intervention

Researchers have investigated the effectiveness of MBIs on a wide range of conditions, including anxiety disorder, depression, chronic pain [65], sleep disturbances [66–68], and fibromyalgia [69]. In 2010, a systematic review and meta-analysis of published randomized controlled trials was performed to examine the effectiveness of MBSR on depression, anxiety and psychological distress across populations with different chronic somatic diseases. Eight total studies were included, and the authors found an overall small effect size of MBSR on depression, anxiety, and psychological distress (Hedge’s g effect size of 0.26, 0.47, and 0.32, respectively) [70]. Another systemic review and meta-analysis was performed in 2013 to examine the effectiveness of MBSR for fibromyalgia syndrome, a chronic condition characterized by widespread pain, fatigue, cognitive disturbances, and sleep disorders. Six studies were included in the analysis, which revealed low quality evidence for short-term improvement in quality of life and pain after MBSR when compared to usual care and active control interventions; no evidence was found for long-term effects of MBSR [69]. Meanwhile, MBB has been found to be efficacious compared to a standard of care sleep hygiene program in improving self-reported symptoms of sleep disturbance in veterans [66] and cancer survivors [67, 68]. When used as an adjuvant treatment for substance abuse in women, MBB reduced self-reported drug cravings, trauma-related thinking, and sleep disturbances [63]. In the studies mentioned above, MBB also improved the following self-reported secondary outcomes: PTSD [66], depression [67], awareness or mindfulness [63, 67], self-compassion [63, 67], and well-being [63, 67].

### Application of the intervention to GWI

MBIs have been investigated as potential treatment methods for alleviating some of the symptoms associated with GWI, after promising results were observed in similar conditions, such as anxiety disorder, chronic pain, and depression. In 2002, subject matter experts recommended that interventions developed for GWI be integrative, however, few integrative treatment approaches have been investigated to date [71]. With CBT showing only a modest benefit on several symptoms of GWI and no significant effect on fatigue, there was a need for additional studies of integrative approaches for the treatment of GWI [49].

Investigators identified MBIs as potential integrative treatments for GWI due to peer-reviewed published clinical trials that indicated that MBIs are associated with small to modest improvements in general symptom severity, sleep disturbance, pain, depression, and anxiety, as well as reduced fatigue among individuals with CFS [72, 73] and analogous conditions [61–63]. Additionally, although MBIs had not been assessed specifically in GWVs with cognitive deficits, some studies indicated that mindfulness training influences attentional and memory abilities [56, 74]; this evidence suggested that MBIs could be particularly well suited for the management of GWI symptoms. Investigators sought to assess whether participation in MBIs, specifically MBSR, as an adjunct therapy would be effective in improving some of the problems experienced by GWVs relative to treatment as usual. Among the 55 GWVs who met the CDC criteria for GWI in the VA-funded study, participation in MBSR, in comparison to treatment as usual, was associated with significant improvements in pain (*F* = 0.33; *P* = 0.049), fatigue (*F* = 0.32; *P* = 0.027), and cognitive failures (*F* = 0.40; *P* < 0.001) when assessed 6 months post-intervention; cognitive failure was the only primary outcome that exhibited a significant reduction at the immediate posttest time-point (*F* = 0.44; *P* = 0.002). Depressive and PTSD symptoms were also greatly reduced for those randomized to MBSR immediately posttest (depression: *F* = 0.22; *P* = 0.050; PTSD: *F* = 0.44; *P* = 0.005), with depressive symptoms maintaining significance at the 6-month post-intervention time-point (*F* = 0.27; *P* = 0.031) [75].

Expanding on MBI research within the GWV population, a randomized phase II trial, funded by the DoD/CDMRP, was conducted to investigate the efficacy of MBB, in comparison to sleep education (SED) control, for improving sleep in GWV [76]. In addition to deployment during 1990–1991, the case definition in this study required the presence of self-reported sleep disturbance and at least two other symptoms of GWI. The Medical Outcomes Study Sleep Scale (MOS-SS) was used to assess improvements in self-reported sleep disturbance as the primary outcome; assessed secondary outcomes included PTSD (PTSD Check List–Military (PCL-M)), depression (The Center of Epidemiological Study-Depression Scale (CES-D)), and fatigue (The Multidimensional Fatigue Inventory (MFI)), among others. Salivary cortisol and α-amylase (SAA) were also measured as biomarkers of sleep disturbance and physiological stress (higher levels indicate more stress and sleep deprivation) pre- and posttreatment and at follow-up. The study was completed by 57 veterans who received three weekly sessions of either SED (*n* = 25) or MBB (*n* = 24) for three consecutive weeks. At follow-up, greater improvements in sleep score [*F*(1,180.54) = 4.04, *P* = 0.046], PTSD [**F**(1,56.42) = 4.50, *P* = 0.038)], depression [F(1,93.70) = 4.44, *P* = 0.038], and fatigue symptoms [*F*(1,68.58) = 3.90, *P* = 0.050] were reported for the MBB group. Although no significant difference was observed in cortisol levels, MBB resulted in a slight reduction in the mean waking level of salivary α-amylase at follow-up [*F*(1,88.99) = 3.78, *P* = 0.055] [69].

### Current and future research in GWI

Currently, there are no registered active clinical trials of MBB for GWI. However, Kearney et al. are currently recruiting for a VA-funded, randomized phase III clinical trial evaluating the outcomes of two different group interventions for veterans with CMI. An anticipated 308 subjects will be enrolled, half of whom will be GWVs who meet the Kansas criteria for CMI, while the other half will consist of veterans with CMI from other periods of service. The study will compare the effectiveness of MBSR and an adapted version of the Chronic Disease Self-Management Program (CDSMP) on pain, fatigue, and cognitive failures in veterans experiencing symptoms of CMI using the Short-Form McGill Pain Questionnaire, General Fatigue Subscale of the Multidimensional Fatigue Inventory, and Cognitive Failures Questionnaire, respectively [77].

### Concordance with the NIH model

Reports of efficacy of MBI in the treatment of symptoms also observable in GWI have led to practice-based clinical trials in GWI patients. There has been no effort to date to link the benefits of MBI to objective markers of the pathophysiology of GWI.

## Coenzyme Q10

### Description of the intervention

Coenzyme Q10 (CoQ10), also known as ubiquinol, is a fat-soluble coenzyme that is present in all aerobic eukaryotic cells. It is partly produced in the mitochondria of the cell, which is also where it is mainly concentrated. CoQ10 plays an essential role in energy production by acting as an electron carrier in the electron transport chain of aerobic cellular respiration. Recent research also indicates that it may act as an antioxidant in the cell membrane, protecting the membrane from ROS-induced damage [78]. CoQ10 deficiency manifests when mutations occur in its encoding genes. Mitochondrial defects can also lead to CoQ10 deficiencies [79]. Clinical symptoms of CoQ10 deficiencies include symptoms of GWI and can include retinopathy, cardiomyopathy, muscular weakness, exercise intolerance, neurological defects, peripheral neuropathy, mitochondrial encephalopathy, and ataxia [59].

### History of the intervention

Researchers have investigated the effect of CoQ10 on a range of conditions, including heart failure [80], cancer [81], statin myopathy [82], and periodontal disease [83]. Using CoQ10 as an adjunctive treatment with standard therapy in a sample of subjects with chronic heart failure, investigators showed that, compared to placebo, daily supplementation with CoQ10 (100 mg, 3 times a day) significantly lowered cardiovascular mortality and incidence of hospital stays for heart failure. CoQ10’s effectiveness in treating other conditions such as cancer, statin myopathy, and periodontal disease is highly debated and not currently supported by research. CoQ10, which is a potent free radical scavenger, has also been investigated as a neuroprotective supplement in rats. Researchers found oral administration of CoQ10 to be associated with a significant increase in cerebral cortex mitochondrial concentration of CoQ10 and a significant increase in life span in a transgenic mouse model of familial amyotrophic lateral sclerosis [84]. CoQ10 is not approved by the U.S. Food and Drug Administration (FDA) for the treatment of any specific medical conditions and is only available as a dietary supplement.

### Application of the intervention to GWI

CoQ10 has been investigated as a supplement for improving general health in veterans suffering from GWI. As mentioned in the introduction, many of the exposures GWVs encountered while deployed to the GW theater are known to function as AChEIs. AChEIs inhibit AChE activity, resulting in the accumulation of neurotransmitters (i.e., ACh) in neuromuscular junctions and synaptic clefts. Accumulation of ACh leads to the overstimulation of ACh receptors and hyperactivity of excitable tissue, which ultimately leads to the depletion of ATP that results in mitochondrial dysfunction [85].

Building on the evidence supporting a role for mitochondrial dysfunction from AChEIs, investigators then sought to assess whether supplementation with CoQ10 would be effective in improving some of the symptoms experienced by GWV. Among 46 GWVs who met the Kansas and CDC criteria for GWI in the DoD CDMRP-funded study, in comparison to a placebo, 100 mg per day of CoQ10 significantly improved physical function and self-rated health symptoms based on a Summary Performance Score (summed results for timed chair rises, walking velocity, and standing balance three ways) and a self-rated health questionnaire (self-rated health as poor, fair, good, very good, or excellent and scored ordinally from 1 to 5), respectively [86].

### Current and future research in GWI

Klimas et al. [87] are currently recruiting for a VA-funded, randomized doubled blinded phase III clinical trial investigating the use of CoQ10 as a supplemental treatment for GWI. An anticipated 200 GWVs who meet the Kansas case definition for GWI will be enrolled in this placebo-controlled clinical trial. Subjects will receive 6 months of CoQ10 (ubiquinol) or placebo supplementation (200 mg, twice a day, for 2 months followed by 200 mg, once a day, for 4 months) with a primary outcome measure of physical functioning and related symptoms as quantified by the 36-Item Short Form Survey (SF-36) [87].

### Concordance with the NIH model

Findings from basic science research on CoQ10 in animals have been translated into clinical trials with human subjects for various analogous conditions. Although there is no FDA approval for the use of CoQ10 as a treatment for any specific medical condition, it has been widely adopted as a dietary supplement and an adjuvant therapy for various conditions. Observations of mitochondrial deficiencies among GWVs and reports of the efficacy of CoQ10 in bolstering mitochondrial function have led to practice-based clinical trials in GWI patients, despite the absence of investigations of the effects of CoQ10 in GWI animal models.

## Mifepristone

### Description of the intervention

Mifepristone is a synthetic selective type II glucocorticoid receptor antagonist, which acts on the hypothalamic pituitary adrenal (HPA) axis. The HPA axis is the neuroendocrine system that controls reactions to stress and regulates many body processes, including digestion, the immune system, mood and emotions, sexual health, and energy storage and expenditure. Dysregulation of the HPA axis has been associated with chronic fatigue syndrome, insomnia, burnout, and memory difficulties [88].

### History of the intervention

Mifepristone is an FDA approved emergency contraceptive [89]. It has an additional accepted medical use as a treatment for high blood sugar associated with Cushing’s syndrome [90]. Moreover, research has shown mifepristone’s success in improving mood and cognition in neuropsychiatric disorders, such as psychotic depression and Alzheimer’s disease [91].

### Application of the intervention to GWI

Mifepristone has been investigated as a potential pharmacological treatment for alleviating neuropsychological complications associated with GWI after the promising results observed in conditions with analogous symptoms, such as psychotic depression and Alzheimer’s disease. One study exploring potential biomarkers of GWI found that plasma cortisol (a type of glucocorticoid) levels differed between GWVs (deployed to GW theater; not necessarily GWI) and “healthy controls” (nondeployed veterans). The findings showed substantial reductions in both basal and metyrapone-stimulated levels of adrenocorticotropic hormone (ACTH) (the stimulator of cortisol production) and a significantly higher cortisol to ACTH ratio (indicating an increased responsivity to cortisol’s negative feedback effects on ACTH production) in deployed GWVs in comparison to controls. The study also found that as the value of the cortisol to ACTH ratio increased, so did the number and severity of reported neuropsychological symptoms [92]. Using these findings as their justification, researchers then theorized that dysregulation of the HPA axis was a potential mechanism underlying GWI.

A subsequent study supported the claim that GWVs had a greater responsivity to glucocorticoids in comparison to controls and found that alterations in neuroendocrine function were associated with deployment to the GW and postdeployment musculoskeletal symptoms. Findings from this study implicated that a glucocorticoid-based treatment may be needed to address the neurocognitive symptoms associated with GWI [93]. Using a dosage strategy borrowed from a study of patients with Alzheimer’s disease [94], researchers conducted a DoD-funded, randomized, double-blind, placebo-controlled trial of mifepristone in GWVs meeting the Kansas case definition for GWI (*n* = 36). Treatment with mifepristone was not associated with improvement in self-reported health status or mental health status, but it did selectively improve verbal learning (*P* = 0.008) in the absence of improvement in other cognitive measures, visual learning, and a global composite measure [95].

### Current and future research in GWI

No other studies have investigated the efficacy of mifepristone as treatment for GWI symptoms to date. Observed abnormalities in HPA axis biomarkers within the GWV population suggest that further research exploring HPA axis dysregulation in GWI is warranted, perhaps first in an animal model of GWI or by using a multitiered intervention strategy as suggested by some research [96].

### Concordance with the NIH model

Reports of the efficacy of mifepristone in the treatment of analogous conditions led to a practice-based clinical trial in GWI patients, despite the absence of investigations of mifepristone in GWI animal models. Although biomarkers of HPA function differ in subgroups of GWVs, one published trial of mifepristone reported predictable changes in biomarkers in response to treatment.

## Acupuncture

### Description of the intervention

Acupuncture is a traditional Chinese medicine (TCM) technique that focuses on the release of blockages in the flow of life-energy through the meridian system via penetration of acupuncture points throughout the body [97]. Acupuncture points, which are used for needle penetration, coincide with trigger points of pain [98] and tender points in FM [99], both of which are concepts that are utilized in Western allopathic medicine.

### History of the intervention

In the 1970s and 1980s, researchers used rat models to show that acupuncture results in the release of endogenous opiate peptides in the brain, which leads to an analgesic effect in the body [100–102]. In more recent years, studies of rat models and functional magnetic resonance imaging of the human brain have identified regions of the brain (limbic, paralimbic, midbrain, and spinal cord) and neurological pathways (excitatory and inhibitory neurotransmitters such as serotonin, dopamine, and norepinephrine) affected by acupuncture [103–109].

In 1997, an NIH consensus panel reviewed over 2000 research studies and recommended the use of acupuncture as an adjunct or acceptable alternative treatment for dental pain and postoperative nausea. The panel also encouraged further research into the use of acupuncture as a treatment for other conditions [110]. Since then, numerous clinical trials have been conducted assessing the efficacy of acupuncture, and it has become more widely practiced in the realm of Western medicine. Today, in addition to the treatment of pain and nausea, acupuncture is used as a complementary or alternative treatment for drug addiction, depression, posttraumatic stress disorder (PTSD), anxiety, fatigue, FM, and musculoskeletal pain. The United States’ military currently has standard protocols in place for use of acupuncture as an alternative treatment for pain associated with traumatic injuries and for PTSD [111, 112]. The VHA also recommends acupuncture as a treatment option for pain.

### Application of the intervention to GWI

Acupuncture has not been studied in GWI animal models. However, given the reported efficacy of acupuncture in treating GWI-like symptoms in civilian populations, in 2013, a randomized phase II clinical trial, funded by the DoD Gulf War Illness Research Program, was conducted to assess the efficacy of acupuncture in reducing pain and improving physical function in veterans suffering from GWI (based on the case definition used for the federal GWI registry) [113]. Due to the lack of a single measure that would address all possible combinations of symptoms experienced by GWI patients, the researchers focused on physical function (to reflect overall health) as their primary outcome of interest. They also evaluated pain as a secondary outcome, given that pain is common among GWI patients. Considering the lack of an adequate placebo for blinded trials of acupuncture and the ethical concerns associated with use of placebos, Conboy et al. assigned one group of veterans (*n* = 52) to acupuncture treatments twice per week for 6 months while assigning a second group (*n* = 52) to 2 months on the waitlist followed by acupuncture treatments once a week for 4 months. Compared to veterans who were waitlisted prior to weekly acupuncture treatments, veterans who received acupuncture twice a week for 6 months experienced a 3.6-point (*P* = 0.04) reduction in pain as measured by the McGill Pain scale and a 9.4-point (*P* = 0.03) increase in physical function measured by the Medical Outcome Survey Short Form 36 physical component scale score (SF-36P).

### Current and future research in GWI

The VA War Related Illness and Injury Study Center (WRIISC) of Washington, DC, in collaboration with Georgetown University, is currently recruiting for a VA ORD funded phase II clinical trial that will evaluate the efficacy of meditation and auricular acupuncture in improving sleep quality in GWI patients [114]. This study was initiated in May 2016 and is expected to be completed by the fall of 2018. The investigators are seeking veterans who were deployed to the Gulf War from 1990 to 1991 and are currently suffering from pain, cognitive impairment, and/or fatigue; they do not have to meet CDC or Kansas criteria for GWI. To date, an estimated 172 participants have been enrolled in the study. No other studies have been conducted on the use of acupuncture for the treatment of GWI.

### Concordance with the NIH model

Findings from basic science research on acupuncture have been translated into clinical trials with human subjects for various conditions. Reports of the efficacy of acupuncture in the treatment of other conditions with analogous symptoms have led to phase I/II clinical trials in GWI patients, despite the absence of investigations of acupuncture in GWI animal models. Although there are no standard guidelines for the use of acupuncture, it has been adopted in clinical practice as an alternative form of treatment for various conditions. Specifically, the VA/DoD clinical practice guideline recommends considering acupuncture for patients with pain-predominant CMI [115].

## Carnosine

### Description of the intervention

Carnosine is an endogenous dipeptide concentrated in muscle and brain tissues [115–118]. Carnosine is involved in several biochemical pathways, but its mechanism of action is not fully understood in all pathways. Nevertheless, numerous studies of cultured cells and rodent models have indicated that carnosine scavenges ROS and reactive nitrogen species (RNS) and acts as an advanced glycation product (AGE) inhibitor, preventing muscle fatigue and neurodegeneration associated with the accumulation of ROS, RNS, and sugars in the body [119–125]. Carnosine is currently not FDA approved for any diseases in the United States, but it is available as a dietary supplement.

### History of the intervention

The neuroprotective effect of supplementation with carnosine or β-alanine (the rate-limiting amino acid in carnosine synthesis) before and/or after injury has been observed in rodent models of traumatic brain injury (TBI) [126], closed head injury [127], intracerebral hemorrhage [128], Parkinson’s disease [129] [130], and ischemic brain damage [131–133]. In all disease models, the neuroprotective effect of carnosine correlated with a reduction in one or more of the following: oxidative stress, inflammatory response, apoptosis of neural cells, and/or blood-brain barrier (BBB) disruption.

Low levels of carnosine and its amino acid subunits have been reported in children on the autistic spectrum [134], and the use of carnosine supplements has been shown to improve cognitive behavior, socialization, communication, and vocabulary in children with autistic spectrum disorders [135]. Chengappa et al. also conducted a preliminary randomized trial to evaluate carnosine as an adjunct treatment for schizophrenia [136]. They observed an improvement in cognitive abilities of the carnosine-treated group in comparison to a placebo-treated group. In 2005, Chengappa et al. also initiated a randomized clinical trial to evaluate the effect of carnosine supplementation on cognitive function in patients with bipolar disorder, but no findings have been reported to date [137]. Carnosine’s potential in improving exercise performance in healthy subjects has been studied extensively as well, but findings have been inconsistent [138]. Lombardi et al., however, did report improved exercise performance in patients suffering from chronic heart failure (CHF) after supplementation with carnosine [139], suggesting that it may help alleviate fatigue in patients with CHF.

### Application of the intervention to GWI

Carnosine levels and functions have not been evaluated in animal models or humans with GWI. However, evidence of neuronal damage and reduced AChE activity observed in rat models of GWI and reports of carnosine’s neuroprotective effects in other diseases arising from neurological dysfunction suggest that carnosine intake may alleviate symptoms of GWI. Thus, Baraniuk et al. conducted a DoD-funded pilot phase I/II randomized, double-blinded clinical trial comparing the efficacy of carnosine as an adjunct treatment for symptoms of GWI among GWVs [140]. Veterans were enrolled in the study if they had already been diagnosed with CFS or fulfilled the GWI case definition based on the CDC criteria (carnosine supplementation group: *n* = 12; placebo: *n* = 13). Outcome measures included activity levels and instantaneous fatigue (measured by actigraphy using ActiWatch Score accelerometer devices), CFS severity (based on self-reported CFS severity scores for fatigue, cognition, sore throat, lymph nodes, myalgia, arthralgia, sleep, and exertional exhaustion), quality of life domains (SF-36), cognition (WAIS-R digit symbol substitution test), pain and tenderness (dolorimetry), and gastrointestinal complaints (Rome II criteria). A significant improvement was observed in cognitive function (*P* = 0.046) and diarrhea associated with IBS (*P* = 0.019) in the carnosine-supplemented group after 12 weeks of supplementation. However, no improvement was reported in other measured outcomes.

### Current and ongoing research in GWI

No other studies have investigated the efficacy of carnosine as a treatment for GWI symptoms to date.

### Concordance with the NIH model

Although extensive basic science research has been conducted on carnosine in animal models of numerous diseases arising from neurological dysfunction, it has not yet been evaluated in animal models of GWI. Knowledge gained from bench research involving carnosine has been translated to phase I/II clinical trials involving several patient populations, including GWI patients. However, it has not yet been translated into practice as a treatment for any condition.

## Conclusions

In 2013, the IOM concluded that no specific treatment for GWI had been found to be efficacious and recommended supportive care that is individualized [17]. Despite federal expenditures of over $500 million on GWI research since 1991, no condition-specific treatment has been discovered for GWI. The case studies illustrate a substantial lack of concordance with the NIH roadmap for research. The case study of CoQ10 is perhaps most adherent to this model due to its stepwise progression from basic science (AChEI mechanism of action, observed mitochondrial dysfunction in GWVs) to bedside (identification of CoQ10 as potentially viable treatment) to a practice-based Phase III clinical trial. The CBT and exercise case studies are perhaps the least adherent. The most significant gap in all GWI intervention research is the lack of GWI-specific basic science research needed to support the rational selection of likely successful interventions.

There is still a lack of understanding about the pathophysiology of GWI. Thus, as illustrated by these case studies, many potential treatments for GWI that have been explored in research studies are existing remedies. While this is not unique to GWI, the selection of these interventions has been justified based on benefits and risks as established in patients suffering from other diseases with seemingly comparable symptoms. The assumption that the analogous conditions have the same, or even similar, underlying mechanisms has not been tested and, given the protean nature of the most common symptoms associated with GWI (e.g., fatigue, pain, and cognitive difficulty), appears naïve. Although this approach may limit the risk of harm to veterans with GWI participating in low-risk intervention trials, it has not succeeded in identifying a treatment for this illness.

There are several factors that may hinder our understanding of the pathophysiology of GWI. Many other conditions have found some success in understanding the underlying pathophysiology by identifying or creating animal models of the condition. Although an animal model of GWI was published as early as 2002 [21], the bulk of advances in the field have been published only recently [23, 24, 33–35, 38, 141–146]. While the developed models aid our understanding of potential mechanisms of disease in GWI, they are not definitive. The IOM cautioned researchers that it may be impossible to develop a comprehensive animal model for GWI [147]. Even if such a model could be developed, it would take a significant amount of resources and effort.

Other factors hindering the development of specific, efficacious treatments for GWI include the extensive list of potential toxic exposures and the inability to objectively quantify the exposures retrospectively. While toxic exposures can be approximated in prospective animal studies, no prospective study of humans could recreate the diverse and highly variable exposures of GWVs. Even populations at risk for individual toxin exposures, e.g., pesticides among farm workers, typically use greater personal protective equipment and keep exposures to safe levels.

Another significant barrier to progress is the lack of consensus about a case definition of GWI. The variation in GWI case definitions from study to study also reduces comparability of results between studies.

Finally, given the diversity of symptoms experienced by veterans with GWI, selecting a primary outcome measure becomes tremendously challenging. As noted in the case studies above, a significant number of clinical trials for GWI interventions conducted to date focused on physical function or overall health status as the primary outcome as opposed to specific GWI-related symptoms. The lack of consensus of primary outcome measures, as well as how to assess symptoms, also hinders comparisons across studies and a synthesis of findings.

Practical factors also present challenges in developing interventions for GWI. Given that GWI only affects a subpopulation of veterans (*n* = 250,000) who are scattered across the U.S., recruitment of GWVs for research is a major challenge. Given the geographically disseminated nature of veterans with GWI, a practice-based research network or clinical research organization model may be best suited for intervention studies in GWI. More extensive use of telehealth technologies to enroll and monitor veterans with GWI may also facilitate recruitment and retention of participants.

The VA and DoD are the two largest sources of funding for Gulf War Illness research. Generally, the DoD has focused its funding on smaller scale studies and capacity building (e.g., consortia, new investigator awards), while the VA has funded projects of various sizes, including the few large multisite studies. Despite replication of studies being a part of VA’s Gulf War Research Strategic Plan 2013–2017, few clinical trials with promising results have been repeated at more than one VA site. Of the interventions we reviewed in this paper, only four treatments (CBT, exercise therapy, CoQ10, and mifepristone) have progressed past a phase II trial. More effort focused on collaborations involving multiple study sites and the replication of study findings may help advance GWI clinical research and lead to more treatments being adopted in practice. The DoD-funded Gulf War Illness Clinical Trials Consortium or the VA Cooperative Studies Program may prove to be effective models for larger clinical trials. Further strategic coordination between the VA and DoD Gulf War research programs, already evident through established frequent public meetings and patterns of funding, may facilitate optimal use of these resources.

There has been little interest from the pharmaceutical industry in developing a treatment for GWI. This most likely reflects the limited number of potential consumers for any pharmaceutical product that may be developed. Considering that development of a drug can cost up $2 billion by the time it is out in the market [148], there seems to be little incentive for pharmaceutical companies to invest in development of a drug for which there is a market of under a million patients. While CMI has been described in the U.S. veterans deployed to Iraq and Afghanistan after 2001 [149–153], the deployment-related exposure profile of these veterans is substantially different from that of GWV, and it is unclear whether the pathophysiology of the symptoms of these two cohorts are the same. If they are demonstrated to be substantially similar, the larger population of affected Americans may enhance the likelihood of broader interest and investment by the pharmaceutical industry.

While not the focus of this review, it is important to note other types of research and development supporting the efforts to identify and evaluate treatments for GWI. These include efforts to establish common data elements for the study of GWI [154]; qualitative research to explore preferences, priorities, and the lived experience of GWVs; health services research to harvest meaningful patterns from the healthcare utilization of GWVs, efforts to develop a more rigorous case definition, and evidence synthesis activities. These activities speak to the practice-based treatment research included in the NIH Roadmap. Similarly, cellular or tissue models and the establishment of data and biorepositories may be useful adjuncts to the goal of identifying efficacious treatments for GWI [155, 156].

The research approach toward GWI resembles that of acquired immune deficiency syndrome (AIDS) prior to the 1980s. While the CDC formally identified the disease in 1982, there are documented cases of AIDS in several different regions of the world as early as 1971 [157]. During the period from the first documented cases outside of Africa in the 1970s until formal identification by U.S. clinicians in 1982, AIDS became known as ‘the silent spread’. Once it was formally recognized, AIDS immediately became a subject of intense focus in the research community as Congress passed bills specifically targeted toward AIDS research and treatment as soon as 1983 [158]. The funding and consistency of funding provided for the AIDS epidemic fostered a period of intense discovery as researchers uncovered, in a relatively compressed timeframe, how the disease was spread, what was causing it, what the virus did to the body, and how to best treat and prevent it moving forward. In fact, within the first decade after its formal recognition, researchers managed to discover both the cause of AIDS as well as develop several FDA-approved treatments. By 2019, the federal budget for HIV/AIDS research grew to $2.6 billion [159].

Like the initial response to the AIDS epidemic, the focus of GWI research in the first 5 years after the war was on defining the scope and manifestations of the condition through epidemiologic investigations. Unfortunately, the course of research and discovery for GWI diverges from the experience with HIV/AIDS. GWI has yet to benefit from a clear definition of the condition, a clear etiology, or an adequate understanding of the pathophysiology. In retrospect, it is apparent that scientific progress in HIV/AIDS research adhered to the NIH roadmap model more closely than GWI research activities have. For these reasons, and perhaps others not explored in this review, including the adequacy and consistency of research funding and institutional support and strategic prioritization of funding in key federal agencies, there is no FDA-approved treatment specifically targeting GWI more than 25 years after its formal recognition.

Arguably, prioritizing GWI research funding on characterizing the pathophysiology of the condition until clear treatment targets are identified could substantially advance research progress and treatment discovery. The next steps might include establishing a clearer, narrower case definition of GWI, creating and validating animal models of GWI, enhancing access to large data and biorepositories, and applying the NIH roadmap (or a similar model) to fund research (including replication of studies). Until then, effective treatments may continue to elude investigators, clinicians, and most importantly, veterans, despite the investment of millions of dollars a year.

## Data Availability

Data sharing is not applicable to this article as no datasets were generated or analyzed during the current study.

## Abbreviations

ACh: Acetylcholine
AChE: Acetylcholinesterase
AChEI: Acetylcholinesterase inhibitors
ACTH: Adrenocorticotropic hormone
AIDS: Acquired immune deficiency syndrome
CBT: Cognitive-behavioral therapy
CDC: Centers for Disease Control and Prevention
CDMRP: Congressionally Directed Medical Research Programs
CFS: Chronic fatigue syndrome
CHF: Chronic heart failure
CMI: Chronic multisymptom illness
CoQ10: Coenzyme Q10
DoD: Department of Defense
FDA: Food and Drug Administration
FM: Fibromyalgia
GW: Gulf War
GWI: Gulf War illness
GWIRP: Gulf War Illness Research Program
GWV: Gulf War veteran
IBS: Irritable bowel syndrome
IOM: Institute of Medicine
ME: Myalgic encephalomyelitis
NCBI: National Center for Biotechnology Institute
NIH: National Institute of Medicine
ORD: Office of Research & Development
PB: Pyridostigmine bromide
PTSD: Posttraumatic stress disorder
QuERI: Quality Enhancement Research Initiative
RNS: Reactive nitrogen species
ROS: Reactive oxygen species
TCM: Traditional Chinese medicine
VA: Veterans Affairs
VHA: Veterans Health Administration
WRIISC: War Related Illness and Injury Study Center
